# Hydration Performance Enhancement Mechanism of Steel Slag-Based Cementitious Materials: Synergistic Regulation of Sodium Silicate and Triethanolamine Complexation

**DOI:** 10.3390/ma19122670

**Published:** 2026-06-22

**Authors:** Li Dai, Feng Chen, Hui Chen, Bin Liu, Minghui Lin, Yi Zhao, Sheng Zeng

**Affiliations:** 1 Jiangxi Provincial Communications Investment Group Co., Ltd., Nanchang 330000, China; dlwhut2012@163.com (L.D.); fengchenjpci@163.com (F.C.); huichenjpci@163.com (H.C.); 2 Jiangxi JPCI Maintenance Technology Group Co., Ltd., Nanchang 330000, China; 3 School of Civil Engineering, Chongqing Jiaotong University, No. 66 Xuefu Road, Nan’an District, Chongqing 400074, China; minghuilin0503@163.com; 4 School of Materials Science and Engineering, Chongqing Jiaotong University, No. 66 Xuefu Road, Nan’an District, Chongqing 400074, China; zsheng@cqjtu.edu.cn

**Keywords:** chemical activation, cementitious materials, mechanical properties, triethanolamine, hydration mechanism

## Abstract

**Highlights:**

- Investigate the synergistic activation mechanism of sodium silicate and triethanolamine in steel slag-based cementitious materials.
- Achieve significant improvement in early-age mechanical properties at low activator dosages (5% Na_2_SiO_3_ + 0.05% TEA).
- Correlate hydration product morphology, microstructural densification and mechanical performance via multi-scale characterization.

**Abstract:**

This study aims to enhance the hydration performance and mechanical strength of steel slag-based cementitious materials via the synergistic activation of Na_2_SiO_3_ and triethanolamine (TEA), solving the early-age hydration and low reactivity of steel slag. The mix is 32% steel slag (SS), 43% blast furnace slag (BFS), 12% desulfurized gypsum (DG), and 13% ordinary Portland cement (OPC). The full factorial design uses Na_2_SiO_3_ (4–6%) and TEA (0.03–0.08%) as composite activators. Mortar specimens were tested for compressive and flexural strengths at 3d, 7d, 10d, and 28d. XRD, SEM, FTIR, and TG revealed the hydration mechanism and microstructure evolution. The results show an optimal dosage of 5% Na_2_SiO_3_ and 0.05% TEA increasing compressive strengths at 3d and 28d by 43.10% and 22.09%, respectively, compared with the control group. This synergy improves matrix compactness, supporting the high-value utilization of steel slag and development of steel slag-based cementitious materials.

## 1. Introduction

Steel slag is a bulk industrial solid waste generated by steel enterprises. In China, the cumulative stockpile of steel slag has exceeded 1 billion tons [1], with an annual production of approximately 100 million tons. Steel production is accompanied by high energy consumption and pollutant emissions [2], resulting in substantial amounts of waste. For every ton of crude steel produced, about 0.96 tons of pig iron is consumed, while 0.2 to 0.4 tons of steel slag (SS) is generated [3]. The comprehensive utilization rate of steel slag is generally low [4]. Not only does stockpiling occupy land resources such as cultivated land and incur ongoing maintenance costs, but the harmful components it contains, such as heavy metals, may also leach into soil and water bodies, causing environmental pollution [5]. Therefore, it is imperative to promote the resource utilization of steel slag.

Steel slag, a by-product of steelmaking, possesses latent hydraulic activity due to its mineralogical composition, including C_2_S and C_3_S as well as calcium aluminates and ferrites [6]. However, its slow hydration kinetics, particularly at early ages, often limit its direct utilization in cementitious systems [7]. Chemical activators are employed to overcome this limitation by accelerating the dissolution of these phases and promoting subsequent hydration reactions [8,9,10]. Na_2_SiO_3_, often used as an alkaline activator, provides alkalinity and soluble silicate species upon dissolution in water, which are essential for the hydration reactions [11]. The high pH environment created by Na_2_SiO_3_ accelerates the dissolution of the glassy phases and crystalline components of steel slag, such as C_2_S and C_2_F [12,13]. Zhang et al. [14] reported that the soluble silicate species then participate in the formation of calcium–silicate–hydrate (C-S-H) gel, the primary binding phase in cementitious materials, and other hydration products like hydrotalcite-like phases. Jiang et al. [15] further noted that the evolution of silicate species from waterglass in alkali-activated slag pastes, particularly at early ages, involves both physical-state and chemical structure transformations, with a portion remaining in liquid form before eventually being consumed to form primary gels; the volume and structure of these primary gels are dependent on the silicate concentration and configuration. The hydration kinetics of steel slag are significantly different from those of traditional granulated blast furnace slag, owing to the heterogeneity of its mineral composition, which is dominated by β-C_2_S and contains tetracalcium aluminoferrite (C_4_AF), free lime, and poorly reactive crystalline phases, as well as an early-formed passive layer [16]. Under alkali-activated conditions, the dissolution of C_2_S and the ferrite–aluminate phases is enhanced; the dissolution of the ferrite–aluminate phases releases aluminum ions into the pore solution, promoting the formation of C-(A)-S-H gel and AFm phases [17]. In alkali-activated cementitious systems, the early reaction pathway is dominated by the rapid dissolution of calcium, silicon, and aluminum phases driven by an alkaline environment, followed by precipitation to form binding gels [18], with the composition depending on the chemistry of the activator and the characteristics of the reactive solid phases. For calcium-rich steel slag, a highly alkaline environment (such as that provided by Na_2_SiO_3_) promotes the dissolution of active sites on the surface of glassy and poorly crystalline phases, increasing the concentration of calcium ions and silicate species in the pore solution, thereby driving the nucleation of C-(A)-S-H gel as the primary binding phase [19].

Triethanolamine (TEA) acts as a complexing agent, significantly influencing the hydration process. TEA is a well-known cement additive that regulates setting time, hydration, and mechanical strength [20,21,22]. Liu et al. [23] indicated that its primary mechanism involves chelating metal ions, particularly Ca^2+^ and Al^3+^, which are released during the dissolution of steel slag phases. Jiang et al. [24] found that this complexation accelerates the dissolution kinetics of aluminate phases (C_3_A and C_4_AF) and, to some extent, silicate phases (C_3_S and C_2_S) in the slag. For instance, Tian et al. [25] demonstrated that 1 wt% TEA accelerates the dissolution of brownmillerite and mayenite, promoting the formation of hydration products. The complexation of Al^3+^ ions by TEA delays the nucleation of C-S-H, thereby regulating the timing of hydration. Furthermore, Jiang et al. [26] observed that TEA facilitates the early hydration of tricalcium aluminate and increases the fine-grained content in steel slag powder, which further aids in the depolymerization of steel slag. The synergistic effect between Na_2_SiO_3_ and TEA is particularly noteworthy. Na_2_SiO_3_ establishes the alkaline environment and provides essential silicate species for C-S-H formation, while TEA precisely controls the dissolution rates of key slag components by chelating Ca^2+^ and Al^3+^ ions [27]. TEA regulates the hydration process by complexing metal ions, particularly Ca^2+^ and Al^3+^, and adsorbing onto active surfaces, thereby altering the dissolution–precipitation equilibrium and nucleation kinetics [28]. In calcium aluminate-rich systems, TEA complexes Al^3+^, increasing the solubility of aluminate species, and, depending on the concentration and sulfate availability, delays or directs the precipitation of aluminum-bearing phases, for example, shifting from ettringite (AFt) to AFm or amorphous aluminum-containing phases [29]. Salha et al. [30] computationally demonstrated that Al^3+^ significantly lowers the energetic barrier for silicate oligomerization through its flexible coordination switching, promoting longer aluminosilicate chain formation under alkaline conditions. Salha et al. [31] further revealed that Al incorporation imparts greater angular flexibility to O-Al-O and Al-O-Si hinges in C-(A)-S-H gels, enabling local conformational stress dissipation and enhancing gel toughness. Tian et al. [25] found that the synergistic effect of high-volume steel slag (40–60%) with blast furnace slag and desulfurized gypsum can form a dense microstructure and effectively improve volume stability. Wang et al. [32] revealed the synergistic activation mechanism of TEA and CO_2_ on steel slag, demonstrating that hydration–carbonation coupling can optimize pore structure and enhance material performance. Yang et al. [33] demonstrated that NaHCO_3_ acts as both a hydration modulator and an internal carbonation promoter in Mg(OH)_2_–NaOH composite-activated high-alumina slag. Furthermore, Nie et al. [34] and Liu et al. [35] emphasize that the evaluation of waste-based binders should consider not only quasi-static strength but also practical limitations.

To prepare highly reactive low-carbon steel slag-based composite cementitious materials and improve the early-age strength of industrial solid waste-based cementitious systems, this study conducted preliminary experiments using the simplex centroid method and constructed a quaternary cementitious system with the following composition: 32% SS, 43% BFS, 12% DG, and 13% OPC. A full factorial design was employed in this study. Nine activation schemes were designed by adjusting the dosage of Na_2_SiO_3_ with a modulus of 2.3, i.e., the molar ratio of SiO_2_ to Na_2_O equals 2.3. (4%, 5%, and 6%), and TEA dosage (0.03%, 0.05%, and 0.08%). A full factorial design was adopted with three Na_2_SiO_3_ dosages (4%, 5%, 6%) and three TEA dosages (0.03%, 0.05%, 0.08%), plus a control group (CG), resulting in ten mix proportions. For each mix proportion and each curing age (3, 7, 10, 28 d), three prismatic specimens (40 mm × 40 mm × 160 mm) were prepared. Thus, the total number of prismatic specimens was 10 × 4 × 3 = 120. Flexural strength was measured on each prism (n = 3 per mix per age), and compressive strength was measured on the two halves obtained from each flexural specimen (n = 6 per mix per age). At the microscale, XRD can identify the crystalline phases formed in the system, FTIR can analyze the functional group information of hydration products such as C-S-H gel and hydrotalcite-like phases, TGA can quantify the content of hydration products and unreacted slag phases [36], and SEM can observe the micromorphology of hydration products and analyze their elemental composition. Based on the above analyses, the evolution law of hydration products and the densification mechanism of the microstructure were elucidated. The core role of TEA in complexing metal ions and the synergistic mechanism of Na_2_SiO_3_ and TEA in breaking the inert surface layer of solid waste particles were revealed. This study provides a pathway for the high-value utilization of industrial solid wastes such as steel slag and effectively promotes the hydration enhancement and mechanical performance improvement of steel slag-based cementitious materials.

## 2. Materials and Methods

### 2.1. Materials

The steel slag-based low-carbon cementitious material used in the experiments was prepared by mixing steel slag powder, granulated blast furnace slag powder, desulfurization gypsum, and Nanhai brand ordinary Portland cement (P·O 42.5R) in specified proportions. The basic chemical compositions of the steel slag powder, granulated blast furnace slag powder, and desulfurization gypsum are presented in Table 1.

To evaluate the basic properties and environmental safety of the steel slag raw material used in this study, its physical and mechanical properties, volume stability, heavy metal leaching characteristics, and radioactivity were tested according to relevant standards. The results are presented in Table 2, Table 3 and Table 4. The steel slag used in this study is a converter steel slag that has been aged for more than one year. The water absorption was determined according to YB/T 4187 [37]; the free CaO content was determined according to GB/T 20491 [38], giving a value of 0.82–1.75%, which meets the standard limit (≤3.0%); and the water immersion expansion rate was determined according to GB/T 24175 [39], giving a value of 0.5–1.5%, which meets the standard limit (≤2.0%). The heavy metal leaching test was conducted according to GB 5085.3-2007 [40], and the radioactivity test according to GB 6566-2010 [41]. Furthermore, after aging, MgO in the steel slag is mainly present in the RO phase (Fe-Mg-Ca-Al composite oxides) with low reactivity, so the risk of expansion caused by MgO is relatively low. No autoclave soundness or long-term dimensional stability tests were performed on the hardened binder in this study. However, based on the low free CaO content, the satisfactory water immersion expansion rate, and the aging treatment, the volume stability of the steel slag can be preliminarily considered acceptable. These test results demonstrate that the steel slag used possesses good volume stability and environmental safety, providing a fundamental basis for its application in cementitious materials.

### 2.2. Experimental Method

The simplex centroid design, abbreviated as SCMD, is a mixture experiment method based on the constraint that the sum of all component volume fractions equals 100% [35]. For a quaternary system comprising SS, BFS, DG, and OPC, denoted as x_1_, x_2_, x_3_, and x_4_, respectively, the design space is a three-dimensional regular simplex, which is a tetrahedron. Seventeen representative points are selected: four vertices representing single components, six edge midpoints representing binary equal blends, four face centroids representing ternary equal blends, one overall centroid representing the quaternary equal blend, and two additional points for error estimation. The construction of mix proportion points is calculated using Equation (1).

(1) $$Y=\sum \beta_{i}x_{1}+\sum \beta_{\mathrm{ij}}x_{1}x_{2}+\sum \beta_{\mathrm{ijk}}x_{1}x_{2}x_{3}{+\beta }_{1234}x_{1}x_{2}x_{3}x_{4}$$

A special quartic polynomial regression model incorporating high-order interaction terms is fitted, with coefficients representing linear, binary, ternary, and quaternary effects [42,43]. Based on preliminary experiments conducted using Design-Expert software 13, 17 design points were established according to the simplex centroid method. The 7-day compressive and flexural strengths were measured, and the mechanical strength data were used to optimize the model.

### 2.3. Test Method and Specimen Preparation

The mix proportions for preparing the 40 mm × 40 mm × 160 mm mortar specimens and their corresponding paste samples of steel slag-based composite cementitious materials were determined based on the results of preliminary single-factor experiments. The quaternary cementitious base materials, namely, steel slag (SS), blast furnace slag (BFS), desulfurization gypsum (DG), and ordinary Portland cement (OPC), were used at a mass ratio of 32:43:12:13. The composite activator consisted of Na_2_SiO_3_ solution (modulus = 2.3) and triethanolamine (TEA). To investigate the effects of Na_2_SiO_3_ and TEA on the hydration process and microstructure of the cementitious system, the dosages of Na_2_SiO_3_ were set at 4%, 5%, and 6% of the total mass of the cementitious base materials, while the TEA dosages were set at 0.03%, 0.05%, and 0.08%, where these percentages refer to the mass of the liquid sodium silicate solution (solid content 43.5%) relative to the total binder mass. The TEA dosages (0.03%, 0.05%, 0.08%) refer to the mass of pure TEA (purity ≥ 99%) relative to the total binder mass. The total water (including the water contained in the Na_2_SiO_3_ solution) was fixed at 225 g, and the total binder mass was 450 g, which resulted in an effective water-to-binder ratio of 0.5. Preliminary tests indicated that Na_2_SiO_3_ showed weak activation at dosages below 4%, while workability decreased at dosages above 6%. TEA was found to effectively regulate the early-age hydration rate within the dosage range of 0.01% to 0.1%. A full factorial design was employed, resulting in nine composite activator groups, with an additional blank control group.

During preparation, the mortar mixture was blended using a JJ-5 planetary cement mortar mixer (Wuxi Jianyi Instrument Machinery Co., Ltd., Wuxi, China). The cementitious binders and standard sand were first dry-mixed for 30 s. Subsequently, mixing water containing the composite activator was added, and mixing continued at a speed of 62 ± 5 rpm for 120 s to ensure uniform dispersion and avoid clumping. The thoroughly mixed mortar was then poured into 40 mm × 40 mm × 160 mm prism molds. The molds were vibrated for compaction using a ZT-96 cement mortar vibrating table(Wuxi Jianyi Instrument Machinery Co., Ltd., Wuxi, China). After molding, the surfaces of the molds were covered with plastic film. The specimens were cured in a standard curing chamber at a temperature of 20 ± 1 °C and a relative humidity of ≥90% for 24 h before demolding. Demolded specimens continued to be cured under the same conditions until the target testing ages.

#### 2.3.1. Mechanical Properties

The mechanical properties of the steel slag-based composite cementitious materials were evaluated on specimens with dimensions of 40 mm × 40 mm × 160 mm. After curing to the target ages, compressive and flexural strength tests were conducted using a computer-controlled electro-hydraulic servo press. The loading rate was set at 0.6 kN/s, complying with the specifications for mechanical property testing outlined in GB/T 17671-2021 [44]. Tests were performed at curing ages of 3, 7, 10, and 28 d to characterize the evolution of mechanical properties.

#### 2.3.2. SEM Testing

To investigate the influence of Na_2_SiO_3_ and TEA incorporation on the micro-morphology of the cementitious materials, samples for SEM observation were prepared with dimensions of 5 mm × 5 mm × 1 mm. The sample surfaces were coated with a platinum layer using a JEOL JFC-1600 auto fine coater (JEOL Ltd., Tokyo, Japan).

#### 2.3.3. XRD Testing

To investigate the effects of Na_2_SiO_3_ and TEA on the mineral composition and phase structure of the cementitious materials, XRD analysis was conducted using a Bruker X-ray diffractometer (Bruker AXS, Karlsruhe, Germany). Samples were crushed, ground, and sieved through a 200-mesh sieve. The powder passing the sieve was evenly spread on the sample holder for testing. The scanning range was 10–80° (2θ), with a scanning rate of 5°/min. Through analysis of the position, intensity, and full width at half maximum of characteristic diffraction peaks, the phase types of hydration products and their crystallinity were characterized. All experiments were performed in triplicate to ensure data reliability.

#### 2.3.4. FTIR Testing

To further elucidate the effects of Na_2_SiO_3_ and TEA on the chemical composition and functional group structure of cementitious materials, tests were conducted using a Bruker FTIR spectrometer (Bruker Optics, Karlsruhe, Germany). The samples were freeze-dried, ground to a micron-scale powder, and mixed with KBr at a mass ratio of 1:100 to prepare pressed pellets. The spectra were collected over a wavenumber range of 4000–500 cm^−1^ at a resolution of 4 cm^−1^. The types of chemical bonds were identified and the hydration reaction process was deduced by analyzing the shifts, intensities, and shape variations of the characteristic absorption peaks.

#### 2.3.5. TG Testing

To investigate the effects of Na_2_SiO_3_ and TEA on the thermal stability and content of hydration products in the cementitious materials, testing was conducted using a simultaneous thermal analyzer (NETZSCH, Selb, Germany). Samples were crushed and ground, and 10–15 mg was weighed into an alumina crucible. The analysis was performed under a nitrogen atmosphere (flow rate 50 mL/min) from room temperature to 800 °C at a heating rate of 10 °C/min. Through analysis of the mass loss rate and thermal effect peaks in different temperature intervals, the contents of hydration products were quantitatively evaluated.

### 2.4. Statistical Analysis

All data are presented as mean ± standard deviation. Flexural strength was measured on three prismatic specimens per mix per age (n = 3); compressive strength was measured on six half-prisms derived from the three flexural specimens (n = 6). This design complies with GB/T 17671-2021 [44] (equivalent to ISO 679:2009 [45]). All comparisons are made against the control group (CG). Normality was assessed using the Shapiro–Wilk test, and homogeneity of variances was verified using Levene’s test, confirming that the data meet the prerequisites for parametric tests. Independent-sample *t*-tests (two-tailed) were used to compare each activator group with the control group (CG).

### 2.5. Semi-Quantitative Analysis of Thermogravimetric Data

A mass loss interval method based on characteristic temperature ranges was adopted for semi-quantitative analysis of thermogravimetric (TG) data, with the aim of estimating the relative amounts of bound water, calcium hydroxide (CH), and calcium carbonate (CaCO_3_) in the hydration products. All TG curves were first normalized to 100% at 50 °C.

Bound water was determined from the mass loss in the 50–380 °C region and corrected to the dry basis at 800 °C, using Equation (2):

(2) $$BW=\frac{m_{50}-m_{380}}{m_{800}}\times 100\%$$

where $m_{50}$, $m_{380}$, and $m_{800}$ are the normalized masses at 50 °C, 380 °C, and 800 °C, respectively.

Calcium hydroxide (CH) content was calculated from the mass loss due to dehydration of Ca(OH)_2_ in the 450–550 °C range. The decomposition reaction is Ca(OH)_2_ → CaO + H_2_O, and the theoretical mass fraction of H_2_O is 18/74 ≈ 0.243. The CH content is given by Equation (3):

(3) $$CH=\frac{\Delta m_{450-550}}{0.243}\times 100\%$$

where $\Delta m_{450-550}$ is the normalized mass loss (on an 800 °C dry basis) in the 450–550 °C interval.

Calcium carbonate (CaCO_3_) content was derived from the mass loss due to decomposition of CaCO_3_ in the 600–750 °C range. The decomposition is CaCO_3_ → CaO + CO_2_, and the theoretical mass fraction of CO_2_ is 44/100 = 0.44. The CaCO_3_ content is calculated using Equation (4):

(4) $${CaCO}_{3}=\frac{\Delta m_{600-750}}{0.44}\times 100\%$$

where $\Delta m_{600-750}$ is the normalized mass loss (on an 800 °C dry basis) in the 600–750 °C interval.

Because the dehydration peaks of C-S-H and AFt overlap in the TG curves, precise deconvolution was not possible; therefore, the above results are semi-quantitative and intended for comparative purposes among different samples. This method is widely adopted as a semi-quantitative approach in thermogravimetric analysis of cement-based materials.

## 3. Results

### 3.1. Material Characterization and Pre-Experiment

#### 3.1.1. Material Characterization

**Figure 1:**
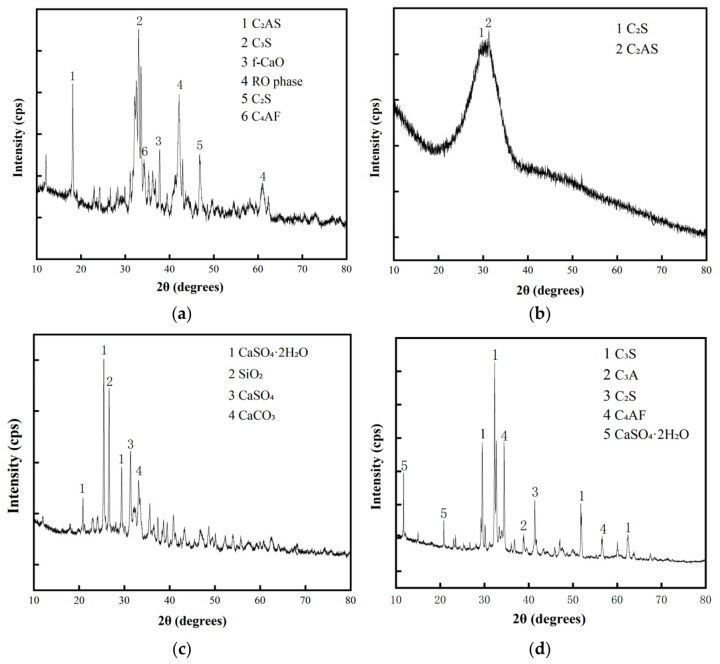
Composition of materials: (**a**) SS, (**b**) BFS, (**c**) DG, (**d**) OPC.

The mineral composition of steel slag (SS), blast furnace slag (BFS), desulfurization gypsum (DG), and cement (OPC) was qualitatively and quantitatively analyzed using XRD, as shown in Figure 1 The main phases in steel slag are tricalcium silicate (C_3_S), dicalcium silicate (C_2_S), the RO phase (Fe–Mg–Ca–Al composite oxides), and free CaO (f-CaO) [46]. The hydrolysis of f-CaO generates Ca(OH)_2_, continuously enhancing the alkalinity of the system. Blast furnace slag exhibits a glassy amorphous structure rich in highly reactive SiO_2_ and Al_2_O_3_. In the strongly alkaline environment provided by steel slag and cement, it hydrates to form substantial amounts of dense C–S–H and C–A–H gels, which fill the internal pores of the system. The main phase in desulfurization gypsum is calcium sulfate dihydrate (CaSO_4_·2H_2_O). The sulfate ions present act as a salt activator for steel slag and blast furnace slag, promoting the formation of ettringite (AFt) to accelerate hydration. This enhances the cementitious activity of both materials and ensures the setting and hardening performance of the cementitious system.

**Figure 2:**
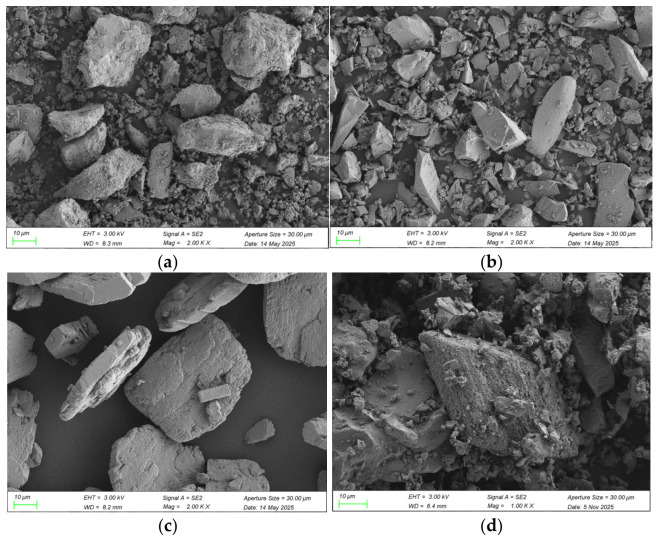
SEM images of materials: (**a**) SS, (**b**) BFS, (**c**) DG, (**d**) OPC.

The SEM of the raw materials shown in Figure 2 SS appears as a brownish powder with irregular chunks, a rough surface, and numerous pores and microcracks, which contain unreacted metal oxides and silicate minerals from the steelmaking process. BFS is primarily composed of a glassy phase, without distinct crystalline boundaries [42]. DG exhibits regular plate-like or columnar crystals with smooth surfaces, clear grain boundaries, and relatively uniform particle size; its main component is CaSO_4_·2H_2_O, which shows high crystallinity. OPC consists of irregularly shaped particles, some of which are partially covered with fine flocculent matter due to slight hydration. It is mainly composed of crystalline minerals such as C_3_S and dicalcium silicate, C_2_S.

The TEA is analytical grade, with a purity of ≥99% in pure form, purchased from Sinopharm Chemical Reagent Co., Ltd. (Shanghai, China). Na_2_SiO_3_ was supplied by a local chemical company, with a Baume degree of 50Be′, which corresponds to a density of approximately 1.53 g/cm^3^, a modulus of 2.3, and a solid content of 43.5%. Deionized water had an electrical conductivity of ≤10 μS/cm.

#### 3.1.2. Pre-Experiment

Using the simplex centroid design, we established the following 17 design points and measured their 7-day compressive and flexural strengths, as shown in Table 5. The mechanical strength data were incorporated into the model, and after optimization and validation, mixture A6 was recommended as the optimal composition. The coefficient of determination R^2^ for compressive strength was 0.991, and that for flexural strength was 0.977, indicating the good predictive capability of the model. Subsequently, the optimal mixture achieved a 28-day compressive strength of 32.6 MPa and a 28-day flexural strength of 7.6 MPa, which were in good agreement with the predicted values. Consequently, the optimal base mixture proportion for the cementitious material was determined to be SS:BFS:OPC:DG = 32:43:13:13.

### 3.2. Analysis of Mechanical Response

**Figure 3:**
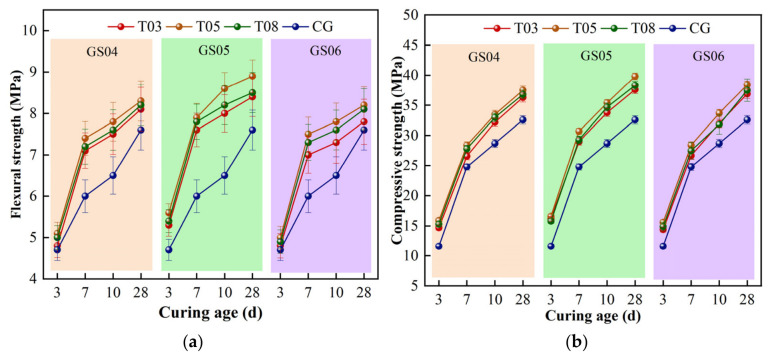
Mechanical properties of all proportions: (**a**) flexural strength, (**b**) compressive strength. The plotted values are arithmetic means, and error bars indicate the standard error of the mean (SEM).

Figure 3 shows that an optimal dosage of composite activator exists. The GS5-T05 formulation, containing 5% Na_2_SiO_3_ and 0.05% TEA by mass of total binder, yields the highest compressive and flexural strengths at all curing ages [47]. The activator effect is non-monotonic. At a fixed TEA dosage, flexural strength ranks from high to low as GS5, then GS04, then GS06.

Insufficient Na_2_SiO_3_ provides weak alkalinity and limits slag dissolution, while excess Na_2_SiO_3_ may form low-polymerized silicate intermediates that coat unhydrated particles and block further hydration. Similarly, among TEA dosages, T05 outperforms T03 and T08. Too little TEA results in poor metal ion complexation; too much TEA migrates to the pore solution and interferes with early nucleation. These results confirm that the composite activator not only accelerates early hydration but also sustains long-term strength development [48].

Table 6 and Table 7 summarize the statistical results of the compressive and flexural strength of the steel slag-based cementitious materials under different mix proportions and curing ages. All comparisons are made against the control group (CG). Normality was verified using the Shapiro–Wilk test (*p* > 0.05 for all groups), and the homogeneity of variances was confirmed using Levene’s test (*p* > 0.05 for all ages), indicating that the data satisfy the prerequisites for parametric tests. The tables present mean ± SD, coefficient of variation (CV), and *p*-values relative to CG. It should be noted that the *p*-values for flexural strength are generally higher than those for compressive strength. This is because flexural strength is more sensitive to microcracks, interfacial transition zone (ITZ) defects, and stress concentration and, thus, exhibits larger data variability than compressive strength. The statistical results reported in these tables fall within the typical range for steel slag-based cementitious materials. Among all mixtures, the GS05-T05 formulation shows superior performance at all ages.

**Figure 4:**
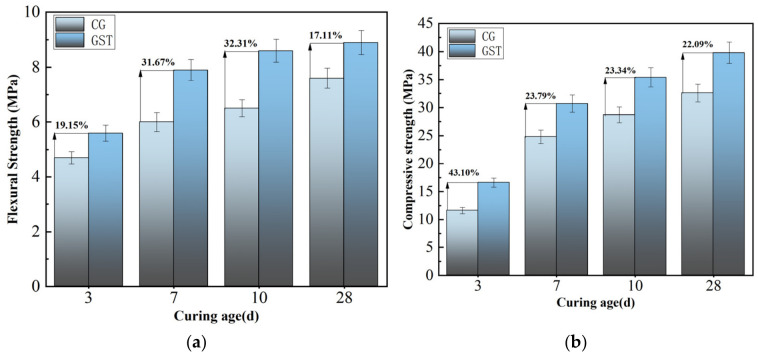
Mechanical properties of the optimal group: (**a**) flexural strength, (**b**) compressive strength. The plotted values are arithmetic means, and error bars indicate the standard error of the mean (SEM).

Figure 4 compares the strength growth rates between the control group and the modified groups. The GS5-T05 formulation exhibits an outstanding mechanical strength improvement. In terms of flexural strength, this formulation reaches 5.6 MPa at 3d, an increase of 19.15% compared to the control group, and further rises to 8.9 MPa at 28d, an increase of 17.11% relative to the control group. In terms of compressive strength, it attains 16.6 MPa at 3d, an increase of 43.10% compared to the control group, and the compressive strength at 28d is 22.09% higher than that of the control group. These results indicate not only that the composite activator effectively promotes the formation of early ductile hydration products and improves the flexural load-bearing capacity of the cementitious matrix but also that the GS5-T05 formulation achieves a synergistic balance between an early activation effect and long-term hydration development.

The performance advantages of this formulation are likely associated with the dual regulatory effects of the composite activator. Na_2_SiO_3_ provides a strong alkaline environment, which is believed to help disrupt the inert oxide layer on the surface of steel slag and blast furnace slag particles, thereby promoting the rapid dissolution of active calcium, silicon, and aluminum ions. Triethanolamine, acting as a complexing agent, may chelate free aluminum ions to avoid local supersaturation of hydration products and simultaneously regulate the crystalline morphology of calcium silicate hydrate gel and ettringite. The synergistic effect of the two activators optimizes the microstructural compactness of the cementitious matrix, ultimately achieving an improvement in the mechanical strength of the cementitious materials [49].

Independent-sample *t*-tests were used to compare the compressive and flexural strengths under different TEA dosages. The combination of 0.05% TEA with 5% Na_2_SiO_3_ achieved a 28-day compressive strength of 39.8 MPa, which was higher than that of the 0.03% (36.3 MPa) and 0.08% (36.8 MPa) groups. Under the present experimental conditions, 0.05% TEA exhibited the highest compressive strength, which suggests that it may be a favorable dosage. Regarding the strength decrease at 0.08% TEA, Zhai et al. [28] suggested that excess TEA may suppress hydration through competing mechanisms such as over-complexation, surface adsorption, and delayed C-S-H nucleation. Furthermore, the microstructural characterizations (XRD, SEM, and TG) of the optimal formulation revealed richer hydration products and a denser matrix, consistent with the mechanical trends. Together with the effect size (absolute strength values and improvement), these observations indicate the existence of an optimal TEA dosage window in this system.

### 3.3. Morphology

**Figure 5:**
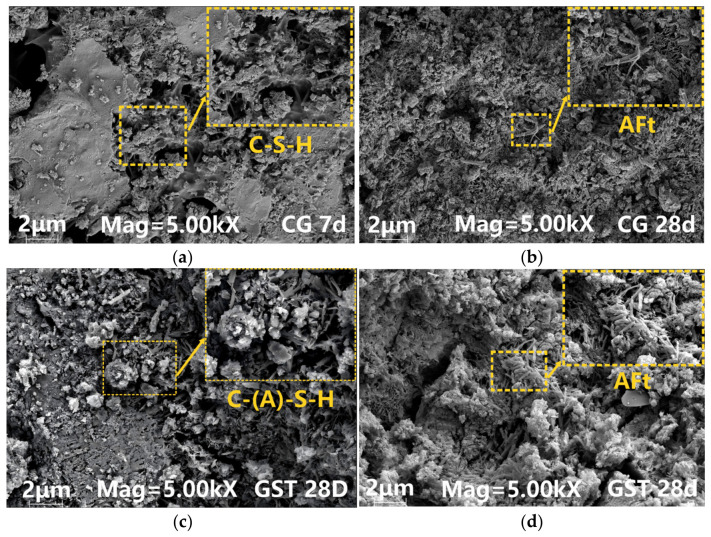
SEM images of hydration products: (**a**) CG at 7d, (**b**) CG at 28d, (**c**) GST at 7d, (**d**) GST at 28d. The scale bar is 2 μm in the main images and 1 μm in the magnified insets (2× magnification).

Figure 5 shows the microstructural features of the cementitious materials. The control group exhibits a relatively loose and porous microstructure, with unhydrated steel slag particles and microcracks visible. Hydration products are mainly plate-like Ca(OH)_2_ and sparse granular phases, leaving internal pores unfilled [50]. By contrast, the optimal formulation group (GS5-T05) exhibits a denser microstructure. At 7 d, abundant circular clustered C-(A)-S-H gel is observed; this morphology may fill interparticle voids and wrap unreacted slag particles, suggesting a possible reduction in porosity. At 28 d, the clustered gel evolves into a dense network and flocculent structure, with dispersed needle-like AFt crystals encapsulated by the gel, which may help form a more continuous structure [51].

The alkalinity of Na_2_SiO_3_ is believed to promote slag dissolution, providing Si, Al, and Ca for the formation of clustered C-(A)-S-H gel, while TEA regulates hydration kinetics by complexing metal ions, helping to control AFt crystal distribution and reduce structural defects. Combined with the semi-quantitative TG data in Table 7 (GST-28d has 6.81% bound water vs. 6.15% for CG-28d, and 4.42% CH content vs. 2.55% for CG-28d), it can be inferred that the synergistic action of the two activators increases the yield and structural integrity of hydration products, leading to a denser microstructure and thereby enhancing mechanical performance.

### 3.4. Composition Evolution

#### 3.4.1. XRD Testing

**Figure 6:**
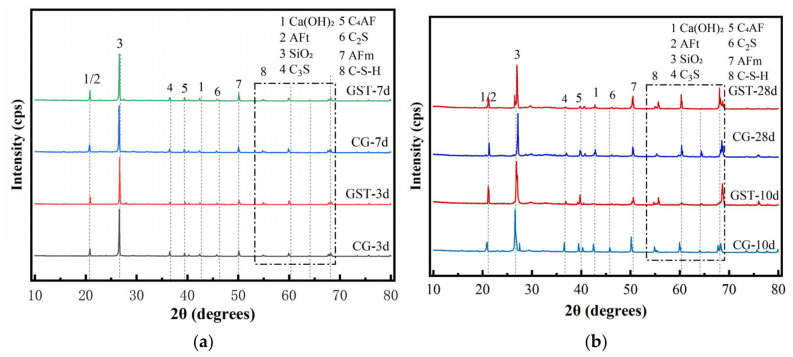
XRD at various curing ages: (**a**) 3d and 7d, (**b**) 10d and 28d. CG is the control group, and GST is the optimal formulation group (5% Na_2_SiO_3_ + 0.05% TEA).

Figure 6 presents the XRD patterns of the CG and GST samples at various curing ages. Characteristic peaks of unhydrated clinker phases including C_3_S, C_2_S, and C_4_AF together with quartz, as well as diffraction peaks of hydration products such as Ca(OH)_2_, AFt, AFm, and C-S-H gel, are identified. With increasing curing age, the intensities of the C_3_S and C_2_S peaks gradually decrease in both groups, whereas the broad amorphous hump corresponding to C-S-H gel becomes more pronounced, indicating continuous hydration and the ongoing formation of amorphous products.

Compared to the CG group, the GST group exhibits weaker unhydrated clinker peaks and stronger C-S-H humps at all ages. At 3 d and 7 d, the AFt peak intensity is higher in GST than in CG, demonstrating that the composite activator effectively accelerates early hydration and promotes ettringite formation. Meanwhile, the Ca(OH)_2_ peak intensity increases more slowly with age in the GST samples, suggesting that Na_2_SiO_3_ in the composite activator consumes part of the Ca(OH)_2_ to participate in secondary pozzolanic reactions, thereby generating additional C-S-H gel. At 28 d, the C-S-H hump of GST-28d is broader and stronger than that of CG-28d, consistent with a higher degree of hydration and greater formation of amorphous products.

Combined with the thermogravimetric results, the GST group shows a notably higher mass loss rate in the 200–380 °C range, which corresponds to the dehydration of C-(A)-S-H gel, and a lower cumulative mass loss in the range of 200–600 °C compared to the CG group. These TG data provide semi-quantitative support that the GST group formed a larger amount of denser C-(A)-S-H gel [52]. Therefore, the phase evolution observed using XRD together with the semi-quantitative TG evidence confirms that the synergistic effect of Na_2_SiO_3_ and TEA significantly promotes the hydration of steel slag and blast furnace slag, enhances the formation of C-S-H gel, and optimizes the phase composition of the cementitious matrix.

#### 3.4.2. FTIR Testing

**Figure 7:**
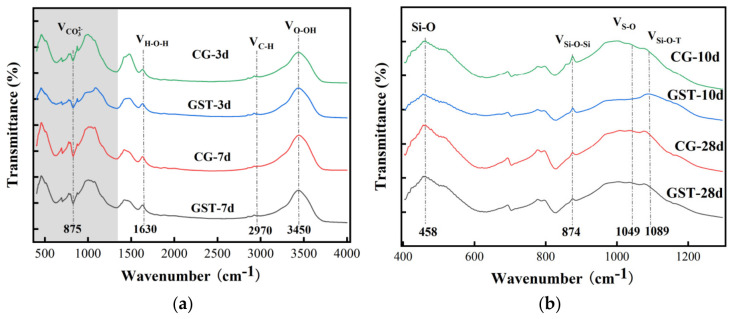

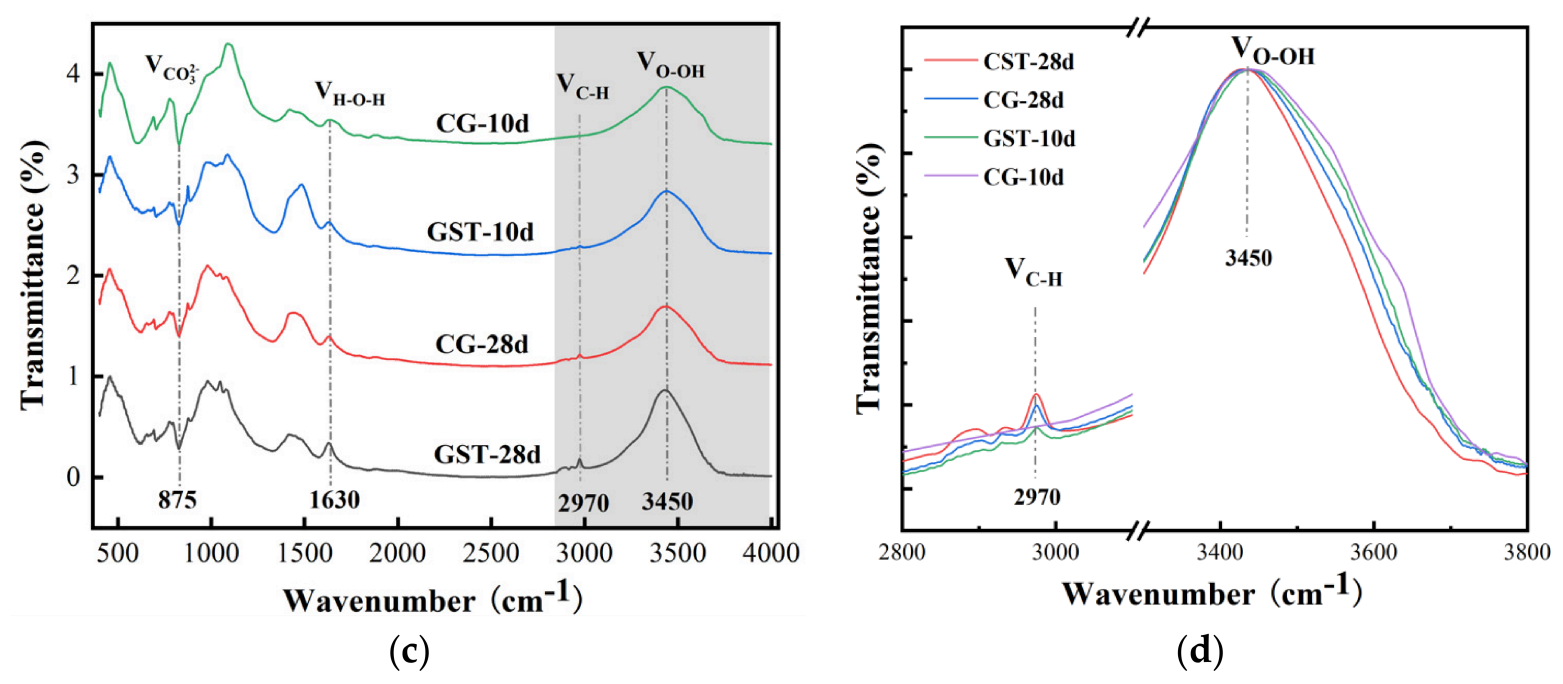
FTIR curves of the cementitious materials: (**a**) 500–4000 cm^−1^ for 3 d and 7 d, (**b**) 400–1200 cm^−1^ in Si–O region, (**c**) 500–4000 cm^−1^ for 10 d and 28 d, (**d**) 2800–3800 cm^−1^ for functional groups. CG is the control group, and GST is the optimal formulation group (5% Na_2_SiO_3_ + 0.05% TEA).

Figure 7 shows the evolution of functional groups and molecular structures in the cementitious materials. The O–H stretching peak near 3450 cm^−1^ is broader and shows lower transmittance in GST-28d than in CG-28d, suggesting that the composite activator may promote the formation of hydration products, thereby increasing bound water and hydroxyl content. The C–H stretching peak of TEA at 2970 cm^−1^ is clearly observed in GST-10d and GST-28d, indicating that TEA may remain in the matrix and potentially interact with hydration products.

In the Si–O bond region, the Si–O bending peak at 458 cm^−1^ shifts left by approximately 4 cm^−1^ (from ~458 cm^−1^ to ~454 cm^−1^) in the GST samples, suggesting increased formation of C-(A)-S-H gel with a possibly denser structure. Combined with the TG bound water calculations (Table 7) and the broader amorphous hump in XRD, the cross-validation by multiple techniques is consistent with a beneficial effect of the composite activator on hydration products. Therefore, it can be inferred that the synergistic action of Na_2_SiO_3_ and TEA may promote the formation of more C-(A)-S-H gel and optimize the microstructure.

### 3.5. Thermal Analysis

**Figure 8:**
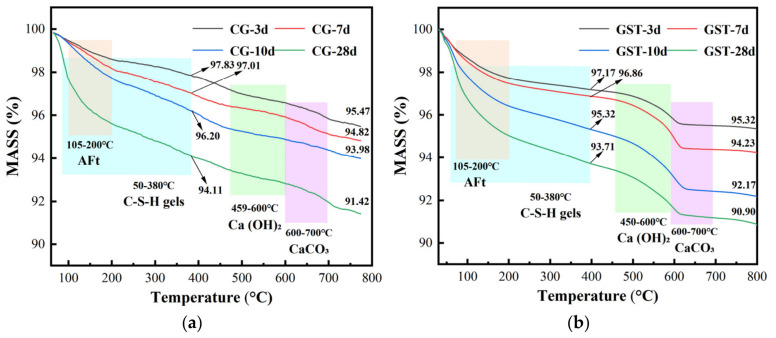
TG curves at various curing ages of 3, 7, 10, and 28 days: (**a**) CG, (**b**) GST. CG is the control group, and GST is the optimal formulation group (5% Na_2_SiO_3_ + 0.05% TEA).

As shown in Figure 8, the thermogravimetric curves reveal the mass loss behavior of different samples. The mass loss of the control group proceeds smoothly and continuously without distinct stages, reflecting the relatively uniform distribution of the hydration products [53]. By contrast, the GST group exhibits three characteristic mass loss stages, indicating a more ordered composition and structure of its hydration products.

Semi-quantitative calculations were performed according to the method described in Section 2.4. The results of bound water, CH content, and CaCO_3_ content for all samples are summarized in Table 8.

The first stage (50–380 °C) covers the removal of crystal water from AFt (mainly at 105–200 °C) and the loss of bound water from C-(A)-S-H gel. The bound water calculations in Table 7 effectively reflect the degree of hydration of the cementitious materials. GST-28d exhibited 6.81% bound water, higher than the 6.15% of CG-28d. This difference indicates that the GST group produced a larger total amount of hydration products, consistent with the improvement in mechanical strength [54].

The second stage (450–700 °C) is mainly related to the decomposition of Ca(OH)_2_ (450–600 °C) and residual calcium carbonate (600–700 °C). The GST group showed higher CH content than the CG group at 7 d, 10 d, and 28 d (e.g., 4.42% compared to 2.55% at 28 d), reflecting the increased alkalinity of the system and higher calcium ion concentration. Furthermore, the CaCO_3_ content of the GST group was consistently lower than that of the CG group (e.g., 1.27% compared to 3.09% at 28 d), indicating the better carbonation resistance of the GST group. This may be attributed to the synergistic activation effect of TEA with CO_2_ [32], which can effectively reduce carbonation. Overall, the carbonation degree of all samples was relatively low and did not substantially affect the analysis of the main hydration products.

Overall, according to the TG curves and semi-quantitative calculations, the residual mass of the GST group at both 380 °C and 800 °C was consistently lower than that of the CG group, indicating a larger total amount of hydration products. Together with the denser microstructure observed using SEM, it can be inferred that the composite activator significantly enhances mechanical performance by promoting hydration product formation and microstructural densification.

## 4. Discussion

This study reveals the synergistic activation mechanism of Na_2_SiO_3_ and triethanolamine (TEA) on steel slag-based cementitious materials. The main conclusions are as follows:

(1) The combination of 0.05% TEA with 5% Na_2_SiO_3_ achieved a 28-day compressive strength of 39.8 MPa, which was higher than that of the 0.03% TEA group (36.3 MPa) and the 0.08% TEA group (36.8 MPa). This formulation also exhibited the lowest coefficient of variation (CV) at all ages, which indicates good data reproducibility. Therefore, under the present experimental conditions, 0.05% TEA can be considered a favorable dosage.
(2) Based on qualitative and semi-quantitative analyses using XRD, SEM, FTIR and TG, the following features were observed. XRD shows higher diffraction peak intensities of the hydration products for the composite activator. FTIR shows a left shift of the Si–O band at 458 cm^−1^ by approximately 4 cm^−1^, which is associated with the rapid formation of hydration products. SEM reveals an intertwined network of clustered gel and needle-like AFt crystals. TG calculations provide semi-quantitative data on bound water, CH and CaCO_3_ content. Based on this multi-technique cross-validation, it can be concluded that the composite activator effectively increases the formation of hydration products, thereby improving mechanical performance [55]. Furthermore, atomistic studies [25,31] indicate that Al^3+^ favors silicate chain elongation under alkaline conditions, and this theoretical calculation is consistent with the trend of our experimental observations.
(3) Compared with single activation methods such as sodium hydroxide, sulfate, and triisopropanolamine, the present system uses low dosages (5% Na_2_SiO_3_ + 0.05% TEA) to overcome the inherent problems of slow early hydration and low strength of steel slag-based binders, significantly promotes the formation of AFt and C-S-H gel, and achieves a denser microstructure.
(4) This study did not test the heavy metal leaching of the cementitious material, delayed expansion caused by free CaO, or long-term volume stability. Long-term durability properties (e.g., carbonation, chemical attack, and freeze–thaw resistance), engineering performance indicators (e.g., workability, setting time, and shrinkage), and environmental benefits (life cycle assessment and hardened material leaching tests) require further quantitative validation. Therefore, the conclusions of this study are mainly limited to hydration mechanisms and microstructural development, and engineering applicability requires further verification.

In summary, the synergistic activation of Na_2_SiO_3_ and TEA significantly improves the mechanical performance of steel slag-based cementitious materials by promoting hydration product formation and microstructural densification.

## Figures and Tables

**Table 1:** Chemical composition of solid waste materials.

Composition	SS (%)	BFS (%)	DG (%)	OPC (%)
CaO	33.26	29.77	30.26	44.49
SiO_2_	6.42	14.25	0.16	9.16
Al_2_O_3_	2.94	7.89	0.07	3.33
TFe	13.27	0.21	0.04	2.50
SO_3_	0.25	1.00	22.33	1.64
Na_2_O	0.05	0.29	-	0.13
MgO	3.66	4.64	0.30	0.91
MnO	2.72	0.25	-	0.09

Note: TFe denotes total iron (as Fe_2_O_3_).

**Table 2:** Basic properties and volume stability of steel slag.

Property	Measured Value	Standard Limit
Apparent relative density	3.51–3.53	≥2.60
Bulk relative density	3.24–3.30	≥2.60
Water absorption (%)	2.16	≤3.0
Free CaO content (%)	0.82–1.75	≤3.0
Water immersion expansion rate	0.5–1.5	≤2.0

**Table 3:** Heavy metal leaching test of steel slag.

Sample	Ba (mg/L)	Cd (mg/L)	Cr (mg/L)	Cu (mg/L)	Ni (mg/L)	Pb (mg/L)
Steel Slag	1.245	0.004	2.488	0.024	0.025	0.191
Standard limit	≤100	≤1	≤15	≤100	≤5	≤5

**Table 4:** Radioactivity test of steel slag.

Sample	Ra Activity (Bq/kg)	Th Activity (Bq/kg)	K Activity (Bq/kg)	Internal Exposure Index (IRa)	External Exposure Index (Iγ)
Steel Slag	7.7	15.5	0.0	0.0	0.1
Standard limit	-	-	-	≤1.0	≤1.0

**Table 5:** Design points of the simplex centroid design and their 7d mechanical strength.

Sample	SS (%)	BFS (%)	OPC (%)	DG (%)	Compressive Strength (MPa)	Flexural Strength (MPa)
A1	38	40	12	10	17.7	4
A2	40	40	10	10	18.8	4.5
A3	30	46	10	14	12.3	3.5
A4	30	43	14	13	13.4	3.5
A5	34	41	15	10	20.6	5.1
A6	32	43	13	12	23.8	6
A7	30	49	11	10	23.4	4.8
A8	34	44	10	12	19.7	5.3
A9	32	47	11	10	20.9	4.8
A10	38	40	10	12	21.4	4.9
A11	35	40	13	12	21.8	4
A12	31	40	15	14	11.8	3.4
A13	35	45	10	10	18	4.3
A14	33	44	13	10	21	4.8
A15	33	43	11	13	19.4	5.4
A16	34	41	11	14	11.6	3.3
A17	30	45	15	10	24.2	5.1

**Table 6:** Flexural strength and statistical results of steel slag-based cementitious materials.

	3d			7d			10d			28d		
	Mean ± SD	CV (%)	*p*-Value	Mean ± SD	CV (%)	*p*-Value	Mean ± SD	CV (%)	*p*-Value	Mean ± SD	CV (%)	*p*-Value
CG	4.70 ± 0.63	13.4	—	6.00 ± 0.96	16	—	6.50 ± 1.11	17.08	—	7.60 ± 1.18	15.53	—
GS04-T03	4.80 ± 0.70	14.58	0.031	7.10 ± 1.05	14.79	0.072	7.50 ± 1.20	16	0.119	8.10 ± 1.31	16.17	0.164
GS04-T05	5.10 ± 0.66	12.94	0.076	7.40 ± 1.00	13.51	0.027	7.80 ± 1.14	14.62	0.081	8.30 ± 1.18	14.22	0.055
GS04-T08	5.00 ± 0.71	14.2	0.108	7.20 ± 1.04	14.44	0.043	7.60 ± 1.19	15.66	0.067	8.20 ± 1.22	14.88	0.126
GS05-T03	5.30 ± 0.65	12.26	0.092	7.60 ± 0.99	13.03	0.133	8.00 ± 1.11	13.88	0.021	8.40 ± 1.16	13.81	0.039
GS05-T05	5.60 ± 0.52	9.29	0.015	7.90 ± 0.84	10.63	0.008	8.60 ± 0.94	10.93	0.013	8.90 ± 0.96	10.79	0.01
GS05-T08	5.40 ± 0.68	12.59	0.048	7.80 ± 1.04	13.33	0.085	8.20 ± 1.15	14.02	0.101	8.50 ± 1.19	14	0.062
GS06-T03	4.80 ± 0.73	15.21	0.147	7.00 ± 1.09	15.57	0.054	7.30 ± 1.23	16.85	0.152	7.80 ± 1.33	17.05	0.025
GS06-T05	5.00 ± 0.67	13.4	0.02	7.50 ± 1.01	13.47	0.116	7.80 ± 1.10	14.1	0.042	8.20 ± 1.09	13.29	0.094
GS06-T08	4.90 ± 0.70	14.29	0.064	7.30 ± 1.06	14.52	0.035	7.60 ± 1.17	15.39	0.138	8.10 ± 1.21	14.94	0.103

**Table 7:** Compressive strength and statistical results of steel slag-based cementitious materials.

	3d			7d			10d			28d		
	Mean ± SD	CV (%)	*p*-Value	Mean ± SD	CV (%)	*p*-Value	Mean ± SD	CV (%)	*p*-Value	Mean ± SD	CV (%)	*p*-Value
CG	11.60 ± 1.35	11.64	—	24.80 ± 2.37	9.56	—	28.70 ± 2.98	10.38	—	32.60 ± 2.92	8.96	—
GS04-T03	14.70 ± 1.63	11.09	0.068	26.60 ± 2.76	10.38	0.036	32.20 ± 3.51	10.9	0.122	36.30 ± 3.25	8.95	0.157
GS04-T05	15.90 ± 1.55	9.75	0.017	28.40 ± 2.59	9.12	0.041	33.50 ± 3.29	9.82	0.074	37.50 ± 3.04	8.11	0.096
GS04-T08	15.30 ± 1.60	10.46	0.083	27.80 ± 2.73	9.82	0.105	33.00 ± 3.43	10.39	0.029	36.80 ± 3.13	8.51	0.131
GS05-T03	16.20 ± 1.65	10.19	0.044	29.00 ± 2.63	9.07	0.079	33.80 ± 3.32	9.82	0.146	37.60 ± 3.06	8.14	0.033
GS05-T05	16.60 ± 1.35	8.13	0.012	30.70 ± 2.29	7.46	0.009	35.40 ± 2.83	8	0.014	39.80 ± 2.61	6.56	0.011
GS05-T08	15.80 ± 1.56	9.87	0.059	29.30 ± 2.53	8.63	0.022	34.70 ± 3.19	9.19	0.088	38.30 ± 2.91	7.6	0.114
GS06-T03	14.40 ± 1.68	11.67	0.135	26.70 ± 2.88	10.79	0.063	32.00 ± 3.59	11.22	0.161	36.90 ± 3.29	8.92	0.047
GS06-T05	15.60 ± 1.51	9.68	0.024	28.40 ± 2.49	8.77	0.091	33.70 ± 3.18	9.44	0.038	38.40 ± 2.86	7.45	0.077
GS06-T08	14.90 ± 1.64	10.99	0.097	27.50 ± 2.70	9.82	0.128	31.80 ± 3.44	10.82	0.052	37.50 ± 3.05	8.13	0.142

Note: Values are mean ± SD. Flexural strength: n = 3 per mix per age; compressive strength: n = 6 per mix per age (derived from three prismatic specimens). *p*-values correspond to activator group vs. control group (CG). Independent-sample *t*-tests were used; no adjustment for multiple comparisons was applied.

**Table 8:** Semi-quantitative hydration product contents derived from TG curves.

Composition	Bound Water (%)	CH Content (%)	CaCO_3_ Content (%)
CG-3d	2.07	2.54	2.32
GST-3d	2.91	2.56	0.75
CG-7d	2.92	1.48	2.39
GST-7d	3.28	3.58	1.35
CG-10d	3.72	2.03	1.90
GST-10d	4.98	4.50	1.75
CG-28d	6.15	2.55	3.09
GST-28d	6.81	4.42	1.27

## Data Availability

The original contributions presented in this study are included in the article. Further inquiries can be directed to the corresponding authors.

## Abbreviations

The following abbreviations are used in this manuscript:

AFm	Monosulfoaluminate (or AFm phase)
AFt	Ettringite (or AFt phase)
BFS	Blast furnace slag
C_2_S	Dicalcium silicate
C_3_S	Tricalcium silicate
C_4_AF	Tetracalcium aluminoferrite
C-(A)-S-H	Calcium (aluminate) silicate hydrate gel
CG	Control group
CH	Calcium hydroxide
FTIR	Fourier transform infrared spectroscopy
GS5-T05	Formulation containing 5% Na_2_SiO_3_ and 0.05% TEA
GST	Optimal formulation group (GS5-T05 group)
Na_2_SiO_3_	Sodium silicate
SEM	Scanning electron microscopy
SS	Steel slag
TEA	Triethanolamine
TG	Thermogravimetry (or thermogravimetric analysis)
XRD	X-ray diffraction
